# Advanced Materials and Devices for the Regulation and Study of NK Cells

**DOI:** 10.3390/ijms20030646

**Published:** 2019-02-02

**Authors:** Guillaume Le Saux, Mark Schvartzman

**Affiliations:** Department of Materials Engineering, Ilse Katz Institute for Nanoscale Science & Technology, Ben-Gurion University of the Negev, Beer Sheva 84105, Israel; lesaux@post.bgu.ac.il

**Keywords:** NK cells, immune signaling, biomaterials, nanodevices, artificial immune synapse

## Abstract

Natural Killer (NK) cells are innate lymphocytes that contribute to immune protection by cytosis, cytokine secretion, and regulation of adaptive responses of T cells. NK cells distinguish between healthy and ill cells, and generate a cytotoxic response, being cumulatively regulated by environmental signals delivered through their diverse receptors. Recent advances in biomaterials and device engineering paved the way to numerous artificial microenvironments for cells, which produce synthetic signals identical or similar to those provided by the physiological environment. In this paper, we review recent advances in materials and devices for artificial signaling, which have been applied to regulate NK cells, and systematically study the role of these signals in NK cell function.

## 1. Introduction

Recent years have seen a great development in novel materials and devices designed to regulate and study living cells. These materials and devices are diverse in their structure, size, chemistry, and functionality, but are all aimed at providing cells with synthetic environmental cues identical or similar to the cues provided to cells by their natural environment, such as extracellular matrix or adjunct cells. These synthetic cues, in turn, stimulate cells as natural cues do, and regulate vital cell functions. Some of these functions such as adhesion, motility, proliferation, differentiation, and death are generic for all cells, while others are exclusive for certain cell types. For instance, lymphocyte immune function, which is tightly regulated by activating, co-stimulating, and inhibitory signals, can be stimulated by an artificial environment that mimics the surface of an antigen presenting cell.

Applications of novel materials and devices for interaction with cells are diverse. Some of these are designed to interact with specific cells in vivo and guide their function for diagnostic and therapeutic purposes. Others are designed to mimic in vitro environmental cues in order to study how these cues regulate cell function. However, since each such material or device usually provides one specific cue, whereas in vivo cells are collectively regulated by numerous cues, the reliability of these materials and devices to reproduce a realistic cell environment is often questioned. Indeed, can we study cells by regulating them with one signal only, and thus neglecting all other signals whose diverse and rich repertoire can never be reproduced by any artificial cell environment, no matter how complex and sophisticated it is? We think that this question was fairly addressed by B. Geiger in his recent review on engineering synthetic cellular microenvironments, who referred to the complexity, diversity, and multifaceted nature of the extracellular environment as a key challenge for the mechanistic and systematic study of cell signaling, and discussed the artificial cell microenvironment as an attractive solution for this challenge [1].

Recent progress in the area of materials and devices for artificial regulation of cell signaling stemmed not only from the emerging needs of bioresearch, but also from dramatic advances in materials and device engineering, especially at the nanometric scale, which took place in the last two decades. Interestingly, the greatest part of nanomaterials and nanotechnology boost originates in the continuous downscaling of integrated circuits according to Moore’s law [2]. In the last two decades, this downscaling had been foreseen to reach its fundamental limit, and this prediction has driven extensive research efforts aimed at exploring new nanomaterials, often envisioned to replace silicon in circuit technology. The most prominent of these nanomaterials are zero-dimensional nanostructures, such as colloidal nanoparticles, fullerenes [3], and quantum dots [4]; one-dimensional nanostructures such as carbon nanotubes [5], inorganic nanotubes [6], nanowires [7], and nanorods [8]; and two dimensional nanostructures such as graphene [9] and its inorganic analogues [10]. Emerging interest in material nanostructuring also led to the development of new nanofabrication approaches, such as nanoimprint lithography [11], block-copolymer lithography [12], dip-pen nanolithography [13], and soft lithography [14]—all allow facile surface patterning with nanometric shapes, and with no need for expensive and complicated equipment. While the future integration of the above-mentioned nanomaterials and nanofabrication approaches into the technology of integrated circuits is still largely questioned, they are broadly used today in numerous niche applications, including biomedical research. The attractiveness of nanomaterials and nanofabrication for biomedical research applications mostly stems from their ability to structure and manipulate biological matter with sub-10 nm resolution, i.e., the size regime of single biomolecules.

One rapidly emerging biomedical application of novel materials and devices is the study of the immune system, with an emphasis on its key players—lymphocyte cells. Lymphocyte immune function is regulated by the immune synapse, in which activating, costimulatory, inhibitory, and adhesion receptors are assembled into a highly regulated architecture [15,16]. The progressive interest in the synapse structure and function has been largely driven by recent advances in immunotherapy. Both immune checkpoint blockade and chimeric antigen receptor (CAR) therapies have shown great potential for cancer treatment, and today’s vast efforts are aimed at the expansion and improvement of these revolutionary therapeutic concepts. Still, a rational design of immunotherapies is only possible upon a profound understanding of how different receptors integrate and coordinate their signals within the immune synapse. To that end, the exact roles of each receptor must be studied individually and in combination with other receptors. Artificial biomaterials and devices provide an ideal experimental platform for such a study, due to their ability to mimic and control distinct signals relevant to cell immune function. Several recent review papers describe, in detail, the broad range of artificial materials and devices designed for synthetic immune signaling [1,17,18,19,20,21,22]. Still, these materials and devices have so far mostly been applied to T cells. At the same time, fewer research works utilized materials and devices to study two other types of lymphocytes—B cell and Natural Killer (NK) cells, although the number of these works is rapidly growing.

**Figure 1:**
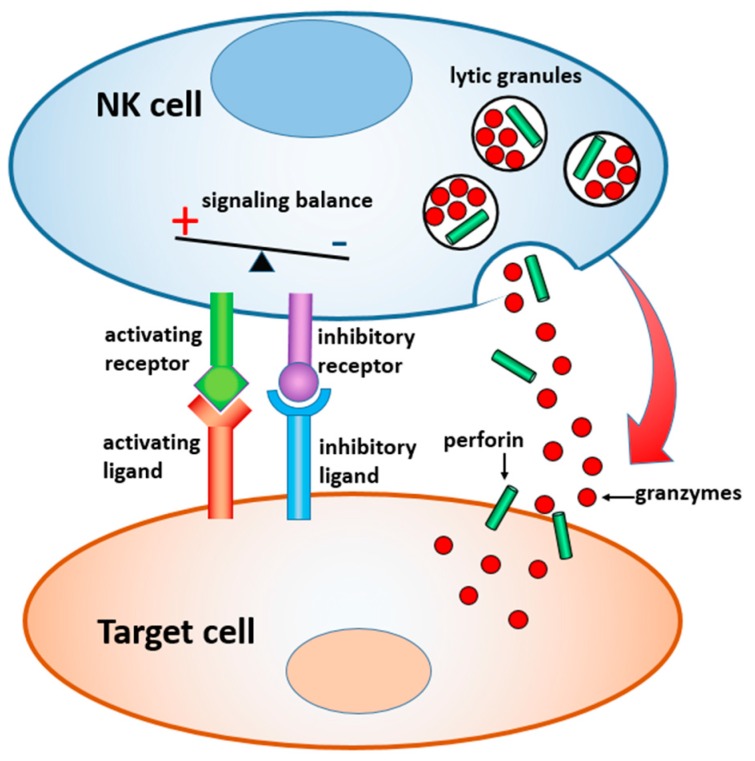
Scheme of the lytic immune synapse formed between a NK cell and a target cell. Degranulation is represented by the red arrow.

**Figure 2:**
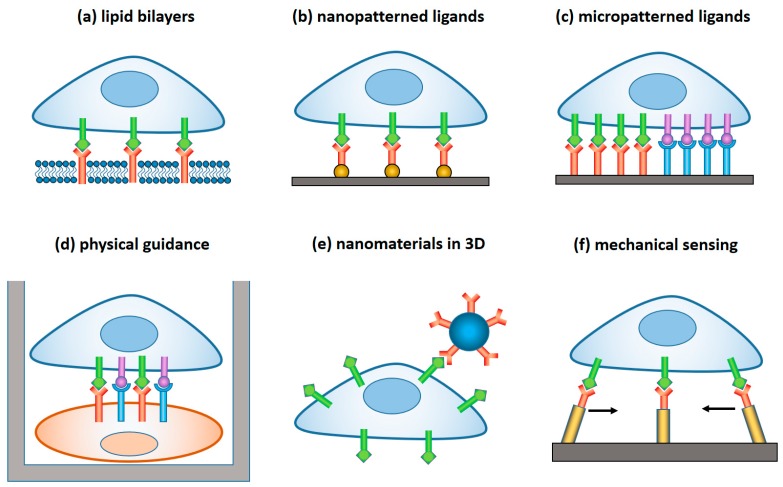
Advanced materials and devices for the study and regulation of NK cells: (**a**) Lipid bilayer with incorporated ligands that mimic the surface of target cells. (**b**) Ligands tethered to a pattern of nanodots that determine arrangement individual receptors onto a fixed 2D architecture. (**c**) Surface with ligand micropatterns that determine receptor clustering. (**d**) Devices for physical guidance and confinement of NK cells, alone as well as in a pair with target cells. (**e**) Nanomaterials for controlled ligand delivery on 3D (**f**) devices for mechanical stimulation of NK cells. Activating or inhibitory ligand-receptor pairs are schematized as in Figure 1.

In this paper, we review materials and devices engineered to regulate and study the immune activity of NK cells. NK cells are the sentinels of the innate immune system, which are distinct from other lymphocytes by their ability to “naturally kill” target cells without earlier sensitization. Activation of NK cells is regulated by a gentle balance between activating and inhibitory signals, which determine whether the target cells will be tolerated or attacked [23,24] (Figure 1). In the latter case, a lytic immune synapse is formed at the interface between the two cells. Granules containing perforin and granzymes are migrated from NK cytosol to the synapse, fuse with the plasma membrane, and release granzymes into the target cells, inducing their apoptosis. Naturally, a broad range of variables related to the structure, composition, and properties of the target surface regulate the signaling pathways within NK cells, and their resulting immune activity. Here, we focus on the materials and devices that either imitate in-vitro the real environment of NK cells in order to study the fundamental mechanisms of NK cell immune activity, or are explored in vivo for diagnostic and therapeutic applications. We classified these materials and derived, by their dimensionality, function, and the way they interact with NK cells, as summarized in Table 1 and Figure 2.

## 2. Planar Lipid Bilayer

The lipid bilayer is among the most basic, and at the same time important form of self-assembly in living organisms. Besides its vital role as the vessel for cells, it serves as a host for a rich repertoire of transmembrane molecules through which cells communicate with its environment. Naturally, lipid bilayers, whose 2D fluidity is similar to that of natural cell membrane, are widely used as the model system for studying processes that take place at the cellular interfaces. First, in vitro lipid bilayers were synthesized more than half a century ago, in the form of a free-standing membrane between two compartments filled with saline solution [25]. Lipid bilayer supported by solid substrate, which are more suitable for cell studies, were introduced in the 1980s [26]. Today, supported bilayers are broadly employed in cell research as model systems for cell interfaces, in particular due the high 2D mobility of lipid molecules and of incorporated transmembrane proteins, as well as the ability to spatially confine lipid bilayers within microfabricated barriers [27].

Early applications of supported lipid bilayers as artificial substitutes for target cells were aimed at inducing the immune response of T cells. As early as 1984, Brian et al. used continuous lipid bilayer to activate cytotoxic T cells cultured with immune spleen cells [26]. Since then, supported lipid bilayers with fluorescently labeled adhesion and activating ligands have been extensively used as an activating environment for the imaging study of the synaptic structure in T cells [28,29].

**Figure 3:**
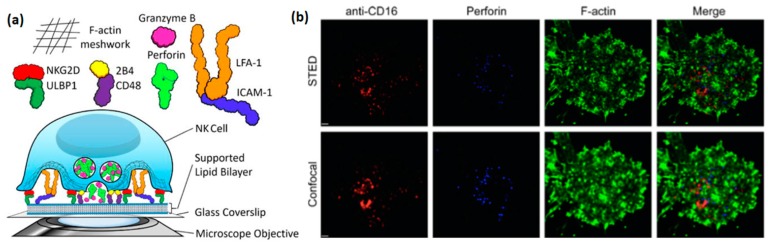
(**a**) Schematic illustration of an artificial immune synapse between NK cell and supported lipid bilayer. Reproduced with permission from Reference [32]. The bilayer includes incorporated ligands recognized by NK cell receptor. The planar interface between the cell membrane and the bilayer enables facile microscopic imaging of the synapse. (**b**) STED imaging of NK synapse on supported lipid bilayer. NK cells were activated on lipid bilayer with incorporated anti-CD16 (red), fixed, permeabilized, and stained with perforin (blue), and anti-(F-actin) (green). Scale bar: 1 µm. Reprinted with permission from Reference [31].

Studying NK cells using supported lipid bilayer (Figure 3a) has arisen relatively recently, but has already provided some highly valuable insight into the structure and function of the NK immune synapse. An early example of such a study is the work of Liu et al., in which lipid bilayers with incorporated ligands for the NK cell receptors NKG2D (CD314), 2B4 (CD244), and LFA1 were used to image activated NK cells by total internal reflection fluorescence (TIRF) microscopy [30]. In particular, this study explored the critical role of ICAM-1, the ligand for LFA-1, for the formation of an organized immune synapse, as well as the regulation of synaptic formation by 2B4, NKG2D, and LFA-1 receptors. Interestingly, a similar synaptic regulation was observed in this study at a synapse formed between two cells, confirming that supported lipid bilayers faithfully reproduce biological processes that occur at the real cell-cell interface. Later, Zheng et al. used stimulated emission depletion (STED) microscopy to image the clustering of activating receptors, perforin, and actin within the NK immune synapse, with a resolution much higher than that allowed by standard confocal microscopy [31] (Figure 3b). This paper, as well as the paper of Bertolet et al. [32], provide detailed protocols for the preparation of supported lipid bilayers, their functionalization with ligands, and imaging of NK cells stimulated into these bilayers.

Ligand-incorporated lipid bilayers can also be used to study the inhibition of NK cells. For instance, Liu et al. in 2012 incorporated into supported lipid bilayer IgG1 Fc that stimulates CD16, and human leucocyte antigen (HLA)-E that engages inhibitory receptors CD94/NKG2A [33]. Using this system, the team elucidated the mechanism of the inhibitory synapse by preventing the buildup of central actin via accumulation of Crk at the synapse center. Similarly, Abeyweera et al. used lipid bilayers with incorporated ICAM, ULBP3 (ligand for NKG2D), and a mutant form of HLA-Cw3 that specifically binds to KIR2DL2, to investigate the role of immunotyrosine-based inhibitory motifs (ITIMs) in the inhibitory pathways on NK cells [34,35]. Interestingly, to study the effect of ITIM on the activating pathways, the group developed a photochemical stimulation for engaging KIR2DL2 during the cell live imaging, which allows imaging of how inhibitory receptors cluster, and concurrently suppress clustering of activating receptors in real time.

## 3. Nanopatterned Ligands

The lipid bilayers described in the previous section allow free 2D movement of the incorporated ligands, and thus provide a convenient platform to study receptor clustering at the cell membrane. An alternative approach to elucidate the role of receptor clustering in cell function is by fixing ligands in controlled arrangements, and thereby creating tightly regulated conditions for receptor clustering. By changing the ligand arrangement and tracking the cell response to these changes, the role of receptor arrangement in various cell functions can thus be elucidated.

Arranging distinct ligands into a controlled fixed pattern is challenging due to their small size, which is usually below 10 nm. One possible approach for such patterning is to chemically anchor ligands to arrays of lithographically fabricated nanodots. The first arrays of these type, which were fabricated by block-copolymer micelle lithography, were applied to study the role of spatial distribution of integrins in the adhesion of fibroblasts [36]. At the core of this approach is the fact that sub-10 nm size of the nanodot ensures that, due to steric hindrance, each nanodot anchors on average one distinct adhesion receptor [37]. Since then, the application of ligated nanodots produced by block-copolymer micelle lithography was expanded to the study of other cells, including T cells [38,39,40].

A few years ago, Delcassian et al. demonstrated the usage of nanodot arrays to study the role of activating receptor distribution in the function of NK cells [44]. To that end, the group patterned glass surfaces with arrays of Au nanodots, whose spacing ranged between 25 to 104 nm, and functionalized the nanodots with CD-16 binding antibodies. NK cells activated on these arrays showed a spreading dependent of the array geometry, with a maximal average spreading on the arrays with the shortest spacing. Interestingly, the group used two types of CD-16 antibodies: 3G8 mAb and Rituximab, which are bivalent and monovalent to CD-16, respectively. However, they found similar responsiveness of NK cell to the array geometry in both cases. The evidence confirms that the number of anchored receptors onto each ligand-functionalized nanodot is determined by the nanodot size rather than by the number of ligation vacancies available on each nanodot.

The great advantage of block-copolymer micelle lithography is that it can easily produce large-area patterns of metallic nanodots, with no need for special equipment. However, as any self-assembly based nanofabrication approach, this lithography is limited to produce hexagonal patterns, whose only degree of freedom is the spacing between the dots, which in turn determines the global nanodot density. Thus, while varying the nanodot arrangement in these arrays can alter cell response, the question will always remain whether this response was determined by the spacing between the dots, or their global density. To address this question, these two geometrical parameters must be deconvoluted, and controlled independently. Such a control was demonstrated by us using nanodot arrays fabricated by nanoimprint lithography [41]. Nanoimprint lithography is based on embossing a polymer resist film with a mold patterned with 3D relief nanometric features. The mold features, which are usually produced by electron-beam lithography, can be shaped and arranged in any arbitrary manner, and easily transferred to the imprinted polymer layer. In that work, we used this advantage to imprint various arrays of sub-10 nm features in a polymer resist, and then transferred the imprinted pattern to arrays of AuPd nanodots by angle-deposition of a metallic shadow mask, plasma etching of the resist through the mask, deposition of AuPd, liftoff, and thermal annealing of the resulting nanodots to controllably shrink them to the desired dimensions [42]. By this method, we fabricated arrays with independently tuned nanodot spacing, global density, and clustering, and used them elucidate the role of each of these geometrical factors in cell adhesion [43].

**Figure 4:**
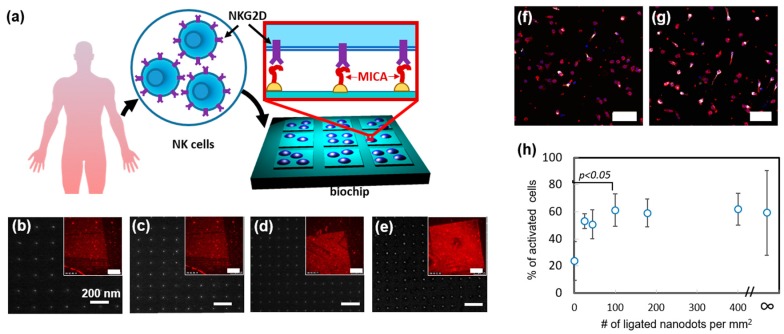
Nanoimprinted chip for the study of the role of the spatial distribution of activating receptors in the immune activation of NK cells. (**a**) Scheme of the chip (**b**–**e**) SEM images of nanodot in different matrices with the periodicity of 200, 150, 100, and 50 nm, receptively. Scale bar: 200 nm. Insets: Florescent image of the matrices functionalized wit MICA and immunostained with AlexaFluor 568 tagged antibody. Scale bar in insets: 50 µm. (**f**,**g**). Fluorescent imaging of CD107a (white) in the NK cells activated on the matrices with 400 nanodots per μm^2^ and 50 nanodots per μm^2^, respectively. The cells were also stained for cytoskeleton (red), nuclei (blue). Scale bars: 50 μm (**h**) % of activated cells vs. the nanodot density. Here ∞ means continues AuPd layer functionalized with MICA, providing the maximal ligand density. Analysis of variance was performed to assess the significant changes in the percentage of activated cells on different matrices. The results were considered significant for *p* < 0.05. Reproduced from Reference [45] with permission from The Royal Society of Chemistry.

Recently, we used nanoimprint lithography to produce devices, which allow it to systematically study how the spatial distribution of activating ligands regulates NK cell function [45]. In that work, we designed and produced a nanochip by nanoimprinting glass with arrays of orthogonally arranged sub-10 nm AuPd nanodots, whose spacing varied between 50 to 150 nm (Figure 4a–e). The chip also contained control areas of and bare glass and continuous AuPd. Remarkably, by forming different arrays on the same nanochip allowed us to quickly and easily assess how NK cells respond to different array geometries in one single experiment and ensure that all the compared cells are exposed to the same environmental conditions except the array geometry. We functionalized AuPd nanodots with MHC class I polypeptide-related sequence A (MICA) ligands, by coating AuPd surface with thiols terminated with nitrilotriacetic acid (NTA), following by NTA chelation with Ni, and conjugation of His-tagged MICA to the formed chelate. To verify that MICA conjugated to the nanodots in a site-selective manner, we stained the chip with anti-MICA antibody and anti-Mouse Alexa 568 and visualized the arrays in fluorescence microscopy.

Using the fabricated nanochip, we first probed how spatial distribution of MICA regulates the spreading of NK cells. To that end, we incubated primary NK cells ono chip surface for 3 h. and measured the average projected area of the cells onto different nanodots arrays, as well as on the control areas. We found, that the arrays of 100 dots per µm^2^ and above stimulated enhanced cell spreading. Next, we studied the role of ligand distribution in the immune activation of NK cells. To that end, we stained the incubated cells with fluorescently tagged antibody of lysosomal-associated membrane protein CD107a, which is a commonly used marker for NK cell degranulation. We found that, whereas the dot distribution on the array had no effect on the average amount of CD107a per cell, it largely regulated the percentage of CD107a positive cells within the overall cell population on the array (Figure 4f,g). Furthermore, we observed that the enhanced population of CD107a positive cells required the same threshold of 100 dots per µm^2^, as the cell spreading did (Figure 4h). These findings clearly show that spatial distribution of activating ligands regulates the spreading and activation of NK cells in a similar manner. Besides providing this important insight into the mechanism of NK cell activation, we demonstrated, in this work, a unique nanotechnological platform that can tune the spatial antigen distribution in an arbitrary manner and allow to independently elucidate the role of each geometry in the function of NK cells.

## 4. Ligand Micropatterns

Nano patterning of ligands with molecular resolution described in the previous section requires unique know-how in sophisticated nanofabrication, as well as specialized equipment, which is usually inaccessible to biologists, otherwise by closely collaborating with nanofabrication experts. At the same time, many studies aimed at understanding the role of receptor clustering in functional cell interfaces, such as the immune synapse, do not require a spatial control of distinct ligands, but can rather rely on patterning ligands within relatively large, often micron-scaled, clusters. Such clusters can be produced, for instance, by microcontact printing, which is also called soft lithography. Microcontact printing is based on mechanic transfer of a molecular ink from a polydimethylsiloxane (PDMS) stamp to a target surface. Since its pioneering in the mid-1990s by the group of G. Whitesides, [14,46] microcontact printing has been very popular in biological research [47], because it is facile, cost effective, and does not require any special equipment.

**Figure 5:**
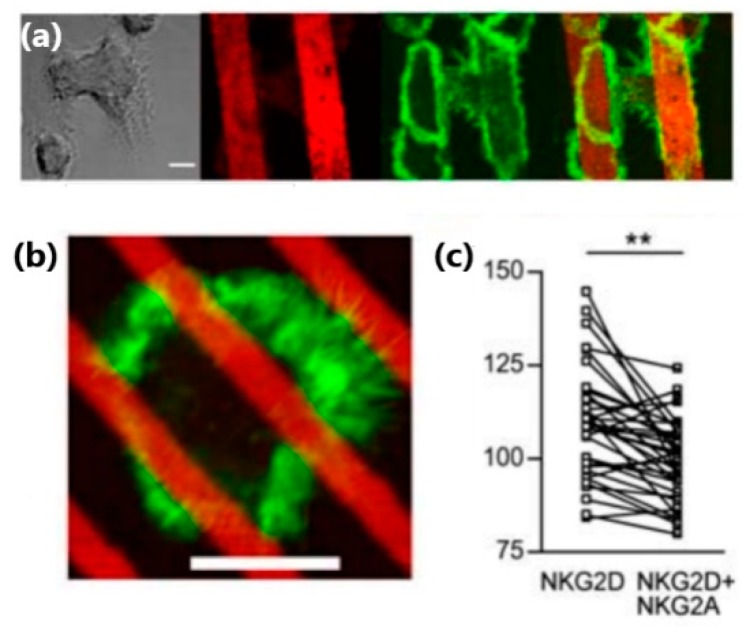
(**a**) NK cells on alternating strips of activating and inhibitory antibodies produced by microcontact printing. NK cells on strips of anti-NKG2D with isotope control mAb (bright filed), antiNKG2D strips (red), f-actin (phalloidin AlexaGluor4888, green), and mixture of the two latter. Scale bar: 25 µm. (**b**) NK cells on narrow lines of anti-NKG2D (red) interspersed with mixed anti-NKG2D and anti-NKG2A. Scale bar: 5 mm (**c**) F-actin distribution in regions of cells in contact with anti-NKG2D stripes or with a mixture of anti-NKG2A and anti-NKG2D (***, *p* < 0.001; *n* = 31 cells, paired *t*-test). Reproduced from Reference [48].

Applications of microcontact printing to the study of NK cells included the fabrication of antigen micropatterns, which bind NK cell receptors in a site-selective manner, and thus control their clustering within the NK cell membrane. For instance, Culley et al. used microcontact printing to produce alternating microstrips of NKG2D antibodies and isotype-matched control mAb, or alternating microstrips of NKG2D antibodies and a mix of NKG2D antibody and inhibitory NKG2A antibody [48]. They found that the spreading and actin polymerization of NK cells plated on these antigen patters were confined to the strips of NKG2D antibody (Figure 5a). Interestingly, this confinement was observed even for microstrips narrower than the cell size, for which one cell could contact a few strips: The intensity of f-actin staining was significantly higher in areas within the cell that directly contacted strips of NKG2D antibody, as compared to the areas that contacted strips with the inhibitory antibodies (Figure 5b,c).

While microcontact printing is a convenient approach for ligand micropatterning, it suffers from two drawbacks. First, each ligand is physisorbed on the surface in a fixed, yet random orientation, and this orientation is not necessarily optimal for the recognition by its cognate receptor. Second, the surface density of ligands patterned by microcontact printing is difficult to control. These drawbacks can be addressed by indirect patterning approach, in which ligands are selectively conjugated to a prefabricated micropattern of chemical functionalities. Here, the conjugation chemistry determines the spatial orientation of ligands, making it uniform through the micropattern; furthermore, the chemical spacer between surface-patterned conjugate and ligands provides the latter with enhanced flexibility, and makes them more available for receptor recognition. Also, the ligand density within the micropattern can be determined by the density of the conjugating functionalities, which is easier to control [91,92,93]. An example of such an approach for micropatterning of NK cell ligands was recently provided by Garrecht et al., who used a piezo-driven ink jet spotter to pattern glass with 200 µm circles of silane molecules terminated with a single DNA strand, and conjugated to these strands complementary strands tagged with avidin, and biotinylated IgG mouse antibody against CD16 receptor [49]. The site-selectivity of the antibody immobilization was verified by immunostaining of the patterned CD16 antibodies with fluorescently tagged donkey-anti-mouse IgG secondary antibody, which revealed a clear fluorescent image of the formed pattern. The patterned arrays were used to stimulate NK cells. In particular, it was found that the varied density of circles in different arrays correlates with the number of stimulated cells detected by the expression of CD107a. This functionalization approach also allowed it to create a pattern of antibodies for 2B4, NKG2D, and their mixture. In the latter case, stimulation of NK cells was found to be more effective than on the patterns of individual antibodies.

Both microcontact printing and ink jet printing are facile and quick patterning methods, however, their resolution is limited to pattern features of a few microns. Smaller features can be fabricated, in principle, by electron-beam lithography or photolithography, however, turning lithographic pattern to a pattern of antigens requires its site selective functionalization with the desired antigens. Whereas such functionalization is possible via various conjugation approaches, it becomes more complicated when the desired pattern is supposed to mimic the molecular diversity of the immune synapse, and thus contain two or more controllably clustered antigens. Recently, we reported an orthogonal approach for site-selective functionalization of a photolithographic pattern with two ligands for NK cell receptors [50]. The pattern consisted of Au disks surrounded by an oxidized Ti film using negative-tone photolithography and etching. To selectively functionalize the pattern with ligands, we first modified the titanium oxide surface using an alkyl phosphonic acid terminated with biotin, to which we attached neutravidin. We also functionalized the Au disks with a NTA terminated thiol monolayer, to which nickel(II) was then chelated. Finally, we selectively attached to avidin and NTA-Ni functionalities two different ligands—one biotin-labelled and another Histidine-tagged, respectively.

**Figure 6:**
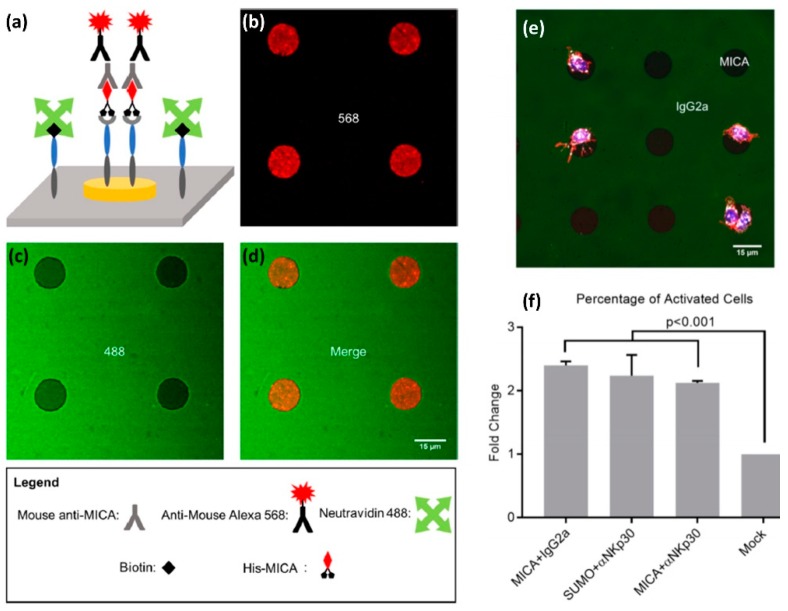
Site-selective functionalization of a photolithographic pattern with two ligands for NK cell receptors. (**a**) Orthogonal functionalization of TiO2/Au surface (**b**) Fluorescent image of Au disks functionalized with His-MICA, and stained with mouse anti-MICA and anti-mouse Alexa 568. (**c**) Fluorescent image of TiO_2_ background functionalized with Oregon Green 488-labeled NeutrAvidin. (**d**) Merging images (**c**,**d**) confirm the site-selective functionalization. (**e**) Primary NK cells stained with Alexa Fluor 555 phalloidin to visualize the cytoskeleton, DAPI for nuclei, and APC-labeled anti-CD107a as a marker for NK cell activation (**f**) Percentage of CD107a positive cells on various regions of the substrates. Analysis of variance and Tukey’s post hoc test were performed to assess the significant changes in behavior. The results were considered significant for *p* < 0.05. *p*-Values are also reported for comparisons of interest. Reprinted with permission from Reference [50].

The antigens we used to demonstrate the site-selective functionalization included MICA, a monoclonal antibody for the NKp30 receptor, and two mock ligands: Murine IgG2a or Small Ubiquitin-like Modifier (SUMO). We prepared four types of bi-functional surfaces: (i) MICA on Au and murine IgG2a on Titanium Oxide, (ii) SUMO on Au and anti-NKp30 on titanium oxide, (iii) MICA on Au and anti-NKp30 on titanium oxide, and (iv) SUMO on Au and murine IgG2a on titanium oxide. We confirmed that the functionalization is site-selective by chemical characterization of different areas with X-ray photoelectron spectroscopy (XPS), as well as by imaging fluorescently tagged antibodies selectively conjugated to the patterned ligands (Figure 6a–d). Furthermore, we incubated primary NK cells on our bi-functional micropatterns and found that the geometry and chemical composition of these micropatterns spatially regulate the motility of the cells. In particular, we found that NK cells incubated on the surface of the type (i) showed higher affinity for MICA-functionalized Au disks than for IgG2a-functionalized background (Figure 6e). Conversely, we found that NK cells incubated on the surface of type (ii) had higher affinity to anti-NKp30-functionalized background than for SUMO-functionalized disks. One possible explanation for these finding is that the cells were washed away during the various steps of the experiments, while only those with a sufficiently strong grip remained. In our case, NK cells formed a strong grip to the areas functionalized with activating antigens.

We also discovered that our bi-functional surfaces can spatially guide the immune activation of NK cells, which we detected by staining CD107a expressed on the surface of activated NK cells with its fluorescent antibody (Figure 6f). We found that, among the cells on the surface covered with activating ligands, more than 80% were CD107a-positive. This number contrasted with less than 40% CD107a-positive cells on the surface covered with mock ligands. Furthermore, we found that the cells activated on the surfaces functionalized with mock-ligands produced on average less CD107a than those activated on MICA or anti-NKp30 surfaces. These results demonstrate the effectiveness of our fabrication and functionalization approach to structure multifunctional surfaces that mimic the molecular diversity and spatial segregation of molecules at the NK immune synapse. Moreover, the key advantage of our orthogonal functionalization approach is in its simplicity and modularity, which stems from the combination of Ni/NTA-His and biotin-avidin conjugations. Both biotin and histidine are very commonly used tags, and the great variety of available proteins and antibodies tagged with histidine and biotin makes our approach the “Swiss knife” of biointerfaces, which can be applied to create endless spatially segregated mixtures of biomolecules.

## 5. Devices for Physical Guidance of Cells

Commonly used techniques for the characterization of cells, such as Enzyme-Linked Immunosorbent Assay (ELISA), Western Blot, and Fluorescence Activated Cell Sorter (FACS), portray collective cell behavior. Yet, it is often necessary to characterize individual cells, especially when there is a need for high-resolution imaging of small features within the cells. Furthermore, cells in culture can be highly heterogeneous, and the discrimination between individual cells is often needed to define cell sub-populations that respond differently to the same environmental cues. Individual cells can be dynamically imaged by high-resolution optical microscopy. Moreover, the automatized stage allows simultaneous time-lapse imaging of numerous individual cells to increase the measurement throughput. However, time-lapse microscopy of individual cells is challenged by the cell motility, because a motile cell can quickly disappear from the field of view of the microscope. This challenge can be addressed by microdevices for trapping individual cells within physical confinements. Whereas the general description of various device concepts, their fabrication approaches, and applications for different cells, can be found in many literature sources (e.g., in References [51,52,53,54]), here we describe the applications of these devices to the study of NK cells.

**Figure 7:**
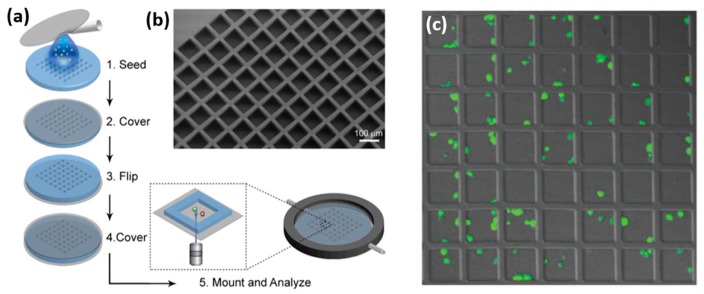
Chip with microwells for confinement. (**a**) Chip fabrication. (**b**) Scanning electron microscope of the chip structure. (**c**) NK cells within the chip after 2 h of incubation. Reproduced from Reference [55].

Microdevices for confining individual NK cells has been recently reported in a few papers by Önfelt and co. In 2010, the group reported PDMS and Silicon microdevices, which contained multiple 80 × 80 µm wells, each capable of accommodating a few NK and target cells [55]. The PDMS devices were produced by casting PDMS mixed with its curing agent onto a photolithographically fabricated master. The resulting microstructured PDMS membrane with seeded NK cells and target cells was clamped between two coverslips (Figure 7). NK cells confined within PDMS devices showed a decreased survival rate after a few hours, probably due to the formation of reactive groups on PDMS surface, or its hydrophobic recovery [56,57]. On the contrary, most of the NK cells confined within a silicon microdevice, which was fabricated by plasma etching, survived for 48 h. Interestingly, it was found that about 30% of cells died much more rapidly than the others. This heterogeneity reflected two cell sub-populations that differed by their mechanism and efficiency of killing. Additionally, the confining of individual pairs of NK and antigen presenting cells enables high-resolution microscopic imaging of the immune synapse formed between the two cells.

Shortly after, the same group reported further applications of microwell arrays for NK cell confinement, this time using microwells of different sizes [58]. They found that 50 × 50 µm wells, which accommodated from one to a few cells, are mostly suitable for their quick and facile evaluation of the heterogeneity of the cell immune response. On the contrary, larger 450 × 450 µm wells, which could accommodate numerous cells, were suitable to study the motility of NK cells, and their interactions with multiple target cells. Later, the devices with small wells were used to categorize individual NK cells into a few sub-populations by their cytotoxic response [59]. In particular, a small but extremely active sub-population of “serial killer” NK cells was detected, in which each NK cell could eliminate a number of target cells, confirming a model by which a small part of NK cells is responsible for tumor elimination. It was also shown that, although the exact distribution of primary NK cells among the sub-populations varies among donors, this distribution has the same qualitative pattern, in which a small fraction of cells kills the majority of target cell [60]. Microwells of similar size were also used by Yamanaka et al. to find a correlation between the activation NK cells and their low motility upon contacting a target cell [61]. In addition, devices with large wells were used for the advanced study of the motility of NK cells, and the dynamics of their contact with target cells [62]. In particular, it was shown that interleukin-2 (IL-2) activated NK cells were substantially more motile compared to resting NK cells, and form much stronger and longer contacts with the target cells. Similar correlations between the motility and cytotoxic activity of NK cells were reported by Merouane et al., who used microwell devices for the automatic profiling of NK cell—target cell interaction, and high throughput time-lapse microscopy of multiple wells [63].

**Figure 8:**
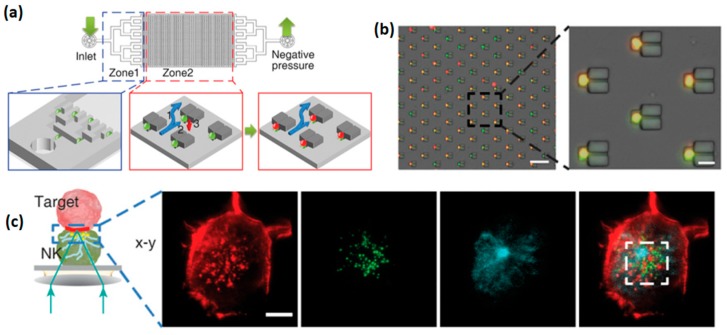
Microfluidic well arrays with microtraps for NK cells. (**a**) Scheme of the microfluidic device (**b**) Merged bright file and fluorescent images after the cell loading into the device; (**b**) red and green channels correspond to K562 and KHYG-1 cells, respectively. Scale bar, 100 µm (left) and 20 µm (right). (**c**) Immune synapse imaged by confocal microscopy at the interface of vertically stacked of NK—target cell pair. The cells are fixed, permeabilized, and stained for F-actin (red), perforin (green), and alfa-tubulin (cyan). Scale bars: 5 µm. Reproduced with permission from Reference [65].

Microwell geometry can be arbitrarily varied to tune the cytotoxic response of NK cells. Recently, Xu et al. demonstrated that NK cells confined within 50 µm wells eliminated breast cancer cells in a more efficient and faster way than NK cells confined within 150 µm wells [64]. Furthermore, they fabricated microwells connected by microchannels that allow NK cell migration between the wells, and showed that the death rate of target cells in these microwells is higher than that in the isolated microwells. Interestingly, microwell shape can also be tuned, sometimes in a sophisticated way, to achieve a desired NK cell trapping device. For instance, Jang et al. fabricated microfluidic arrays of microwells that trap pairs of NK cells and target cells in a vertical orientation [65]. Such orientation facilitated high resolution imaging of the immune synapse formed within the horizontal plane between two cells (Figure 8). For an effective cell confinement, microtrapping features were added to the wells, resulting in the simultaneous capturing of 3000 cell pairs with a loading efficiency of 70%, which is more than twice higher than that achieved for a simple microwell array. Another sophisticated design of microwell for the confinement of NK cells was recently demonstrated by Joon et al., who fabricated a microfluidic device containing parallel microchannels, each with multiple traps for individual cells [66]. Each trap included a branch flow path that allowed the liquid, but not cells, to flow through. Similar to the previously described devices, this microfluidic platform allowed an effective discrimination of the studied NK cells to “weak’ and “strong” sub-populations; the latter consisted of serial killer cells responsible for the elimination of the majority of the target cells. Furthermore, weak and strong NK cells were selectively retrieved using a laser capture microdissection (LCM) system, to allow the downstream mRNA sequencing of each cell group. The selective RNA analysis allowed to elucidate a possible link between the cytotoxicity of NK cells and the overexpression of adhesion molecules, such as ICAM1, and certain genes involved in cytolysis.

Combining trapping microdevices with external forces for the manipulation of NK cells can further facilitate the efficiency of cell separation, or clustering, depending on the need of a specific experiment. For instance, Christakou et al. used standing ultrasonic waves to position NK cells and target cells in microwells [67]. In particular, it was shown that ultrasound could synchronize the formation of NK cell—target cell contacts in multiple wells. Later, the same authors used standing ultrasonic waves to produce controlled 3D cultures of cells in microwells, and monitor the docking and infiltration of NK cells toward tumor cells [68]. This methodology allowed for the comparison of the rate of NK cell lysis to the rate of tumor growth, in order to find the minimal number of NK cells needed to eliminate tumors of different sizes and shapes.

In addition to cell trapping, aimed at tracking their cytotoxic response, microdevices can be designed to study the motility of NK cells. In this case, rather than confining a cell in a limited space, the device should guide its movement along a defined path. One example of such a device was recently demonstrated by Mahmood et al., who realized a microfluidic device that can track, at the single cell level, the migration of murine NK cells along a gradient of chemokines, which are produced in vivo by dendritic cells [69]. In particular, they found that these chemokines induced a high level of chemotactic movement of IL-2-activated NK cells within the microfluidic device and identified CXCR3 as a key chemokine receptor that regulates the migration of NK cells. Besides these findings, the demonstrated microfluidic device provided a new ability to systematically study migratory responses of NK cells to their interaction with antigen presenting cells, including but not limited to dendritic cells.

## 6. Nanomaterials for the Interaction with NK Cells in 3D

Thus far, we have discussed surfaces that are micro and nano-structured with chemical and physical signaling functionalities. Naturally, these surfaces mimic the environment of NK cells in 2D and provide their signal only to the contacting parts of the cells. To introduce stimulating functionalities in 3D in a regulated fashion, e.g., through controlling their clustering, such functionalities can be delivered to cells by nanometric structures. These structures can be chemically synthesized from the bottom-up with a size, shape, and chemical composition controlled at an atomic level, and can be chemically functionalized, e.g., with stimulating ligands for specific interactions with cells in vitro and in vivo. Besides targeted molecular delivery, some nanostructures, such as quantum dots, are broadly used for imaging, due to their strong fluorescent emission. The literature on the interaction of bottom-up synthesized nanostructures with immune cells is quite broad [70], and herein we focus on a few recent examples relevant for NK cells.

One example for the targeted delivery of activating ligands was recently reported by Loftus et al. who used flakes of graphene oxide functionalized with CD16 antibodies to stimulate NK cells in vitro [71]. The flakes were sized between 50 to 300 nm, and each of them accommodated a limited number of antibodies controlled by the flake size. Thus, the antibodies were delivered to NK cells in clusters that mimic the ligand clusters expressed on the surface of antigen presenting cells. It was found, that functionalized flakes of graphene oxide effectively stimulated NK cells, and led to the degranulation of their cytolytic granules and secretion of IFN-γ. Furthermore, it was found that this stimulation largely exceeded that produced by the same amount of antibodies introduced to NK cells as dissolved freestanding molecules. These results emphasize the importance of receptor clustering in the activation of NK cells, and mirror the NK cell response to previously described surface-nanopatterned ligands [44,45].

Ligand-functionalized nanomaterials can also be used for the imaging of NK cells. Lim et al. suggested to use quantum dots to track NK cells used in cancer therapy [72]. For this purpose, they labeled human NK cells with quantum dots functionalized with CD56 antibody. They found in vitro that the viability of NK cells, their production of IFN-γ, and their cytolytic activity, were not significantly affected by their conjugation with quantum dots. Furthermore, in vivo tests done on mice showed that labeling NK cells with quantum dots did not affect their anti-tumor therapeutic efficiency, but at the same time allowed effective near-infrared imaging of the tumor tissue. Additionally, NK cell can be conjugated with nanomaterials for both imaging and immune stimulation. Jiao et al. conjugated NK cells to Au nanoparticles functionalized with CD2 antibody, to enhance the quality of their in vitro computerized tomography (CT), and simultaneously to enhance their in vivo activity against neuroblastoma and melanoma cells [73].

**Figure 9:**
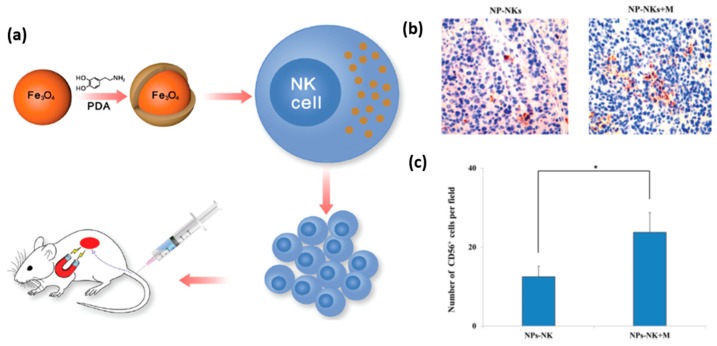
Functionalized nanoparticles for magnetically guided delivery of NK cells. (**a**) Scheme of the entire experimental setup. (**b**,**c**) Distribution and quantitative analysis of the particle labeled NK cells administered by intravenous injection and accumulated at the tumor site after 12 h, with and without magnetic field (Mag: 200×; * *p* < 0.05). Reproduced from Reference [74].

A promising application of nanomaterials conjugated to NK cells is targeted delivery of the latter to tumor tissue for immunotherapeutic purposes. In general, tumor tissues create an environment that challenges the recruitment of NK cells, and thus weakens their immunological efficacy. To overcome this obstacle, and enable an effective recruitment of NK cells to the tumor site, Wu et al. proposed to label NK cells with iron oxide nanoparticles functionalized with dopamine [74]. The magnetic nature of these nanoparticles allowed their guidance towards the tumor site by an external magnetic field, which can be applied using an invasive magnetic device (Figure 9a). It was shown, that dopamine labeling increased their in vitro and in vivo antitumor activity. Specifically, labeled NK cells driven by magnetic field showed increased infiltration into the tumor tissue (Figure 9b,c). This elegant manipulation of NK cells aimed at increasing their antitumor efficiency presents a very promising prospect for future therapeutic applications of nanoparticles. Interestingly, magnetic nanoparticles can also indirectly facilitate the recruitment of NK cells into a tumor. Recently, Park et al. prepared biocompatible polymeric microspheres, and incorporated IFN-γ and iron oxide magnetic nanoparticles into them [75]. The role of IFN-γ was to induce the release of chemotactic cytokines, such as CXCL10, from the tumor, and by this, to stimulate NK cell recruitment. The role of magnetic nanoparticles was to allow MRI imaging of the microspheres, in order to verify their proper delivery to the tumor site and to determine their long-term concentration and distribution in the body. The group used a VX2 rabbit model to demonstrate the in vivo delivery of the microspheres into a liver tumor using a catheter guided by X-ray digital subtraction angiographic (DSA). Using immunohistochemistry, they observed that the loaded microspheres stimulated a substantial increase in NK cell recruitment and infiltration into the tumor.

While biomedical applications of nanomaterials have burgeoned, and their side effects, such as toxicity, must be carefully examined. When nanomaterials are introduced into the body for diagnostic or therapeutic purposes, their potential impact on the immune system remains largely unclear. Several recent studies investigated a possible toxic effect of nanomaterials by exposing them to NK cells in vitro. Müller et al. studied the effect of Ag nanoparticles on the viability and function of primary NK cells, before and after their stimulation with the viral mimetic polyriboinosinic-polyrocytidylic acid [76]. It was found that Ag nanoparticles reduced the viability and cytotoxicity of virally stimulated NK cells and enhanced their expression of the inhibitory receptor CD159a. Additionally, the exposure of NK cells to Ag nanoparticles, as well as to Ag^+^ controls, lowered the expression of the activating receptors CD335 and CD16, and enhanced the expression of the activating receptor CD314. These modifications of NK cell function and phenotype were defined as a potential risk of uncontrolled changes in human immune response. In a similar study, Alam et al. elucidated the effect of acid-functionalized carbon nanotubes on NK cells in vitro and in vivo [77]. It was found that carbon nanotubes significantly decreased the recovery of NK cells, increased their apoptosis, increased their generation of reactive oxygen species, causing poor mitochondrial health and loss of cellular integrity. These studies demonstrate the potential toxicity of nanomaterials for the immune system, which is still largely unexplored, and must be studied in detail to allow their safe medical applications.

## 7. Nanodevices for the Mechanical Studies of NK Cells

Interaction of cells with their environment has been mostly studied in the context of inside-out and outside-in biochemical signals. However, NK cells, as any other cells, are also physical objects, and therefore obey the laws of physics. Cells sense physical properties of their environment by applying mechanical forces on the environment, and transducing the resulting mechanical stimuli into biochemical signals [94,95]. These stimuli determine basic cell function, such as adhesion, motility, proliferation, differentiation, and death [95]. In addition, it is becoming increasingly clear that lymphocytes use mechanical forces to discriminate between healthy and ‘ill’ (e.g., virus-infected or tumorous) cells [78,96]. Furthermore, immunoreceptors recognize antigens under mechanical load to discriminate between high-affinity and low-affinity antigens [79]. Today, the role of mechanical forces in cell immune function, and in particular in the structure and dynamics of the immune synapse, is a subject of extensive research.

It should be noted that detecting mechanical forces in cells is challenging because these forces span over a small, often nanometric length scale, and have very low magnitude down to picoNewtons. Optical traps [80,81], micropipettes [82], and atomic force microscopy [89,90] have been used detect such forces, however they do so only at a single point of the cell membrane. Traction force microscopy based on measuring the dislocation of fluorescent beads in a hydrogel can map forces of an entire cell [91,92,93], however, the exact bead movement is hard to assess since the beads are distributed randomly, and their resting position is unknown. Furthermore, the analysis of the bead displacement requires complicated force calculations based on elasticity theory [88]. Alternatively, forces applied by cells can be mapped by seeding cells onto an array of elastomeric micropillars for cell spreading, whose spatial resolution is limited, however, to the micron-scale. All the above-mentioned methods have been used in recent years for the study of mechanosensing and mechanotransduction in lymphocytes, which, however, has been limited to T cells and B cells [78,89].

**Figure 10:**
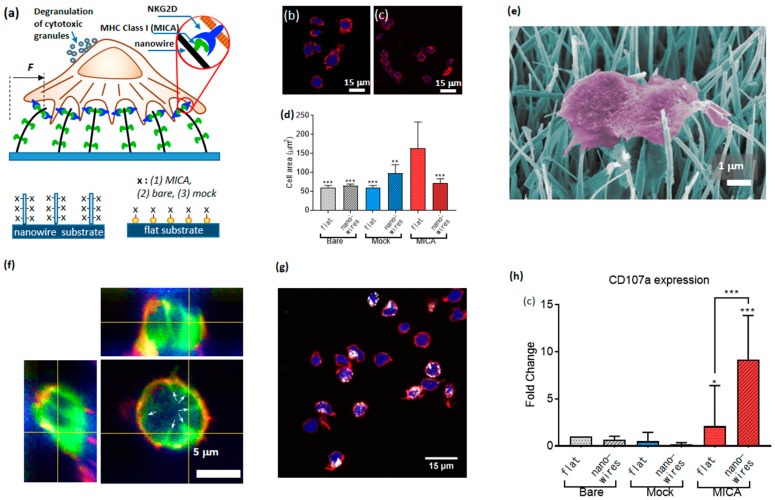
Nanowire platform for the assessment of NK cell mechanosensing. (**a**) Cartoon of the experimental setup, and 6 conditions used to assess NK cell mechanical activity. (**b**,**c**) NK cells spread on flat MICA and nanowire-MICA surfaces, respectively, (**d**) Quantification of NK cell spreading on the tested surfaces (** *p* < 0.001, *** *p* < 0.0001) (**e**) Scanning electron microscope image of NK cell applying forces into adjacent nanowires. (**f**) *Z* stack of confocal microscopy of NK cells with tagged membrane onto MICA-functionalized, bare, and mock-functionalized nanowires, respectively. Cell membrane (green) and actin (red). The white arrows point onto the projected invaginations of the nanowires in the cell membrane. (**g**) NK cells stimulated on MICA-functionalized nanowire. Here, CD107a expression was quantified by measuring the fluorescence intensity of the APC-labeled anti-CD107a (in white). (**h**) Percentage of CD107a positive NK cells on different surfaces normalized surfaces with bare nanoparticles (* *p* < 0.05, *** *p* < 0.0001) Copyright Wiley-VCH Verlag GmbH & Co. KGaA. Reproduced with permission [90].

The first evidence of mechanosensing in NK cells was recently provided by Barda-Saad and co., who showed that NK cell response is mediated by actomyosin retrograde flow [97]. Shortly afterwards, the authors of the present review provided a direct evidence for mechanosensing of NK cells [90]. For this purpose, we developed a novel platform for the study of the cell mechanical activity, which was based on nanowires—quasi 1D nanostructures whose aspect ratio can exceed a few orders of magnitude. We grew dense arrays of vertical nanowires with a diameter of 50 nm and length of 20 µm, using chemical vapor deposition, and seeded primary NK cells on top of the arrays. The ultra-high compliance of the nanowires to the forces applied by NK cells provided mechanical stimuli for NK cell activation. In addition, we provided NK cells with biochemical stimuli by functionalizing the nanowires with MICA ligands. To that end, we coated the nanowire surface with NTA terminated thiols, chelated Ni to the NTA moieties, and conjugated to the formed chelate His-tagged MICA. To study the effect of the mechanical and biochemical stimuli separately, and in combination with each other, we plated NK cells on nanowires and on control flat surfaces covered with Au nanoparticles; in each case, we used three types of functionalization: Bare surfaces, MICA, and mock ligands (six conditions altogether, Figure 10a).

As the first figure of merit for the effect of nanowire on the function of NK cells, we assessed how both the nanowire environment and the presence of MICA ligands affected NK cell morphology. To that end, we measured the area of cells after 3 h. of incubation on MICA-fictionalized nanowires, as well as on other control samples. We found that flat surfaces stimulated enhanced spreading of NK cells (Figure 10b–d). This finding mirrored our previously published results [50]. Concurrently, nanowires, including those functionalized with MICA, could not stimulate substantial cell spreading. A closer look at the cell morphology using scanning electron microscope revealed that NK cells catch adjoining nanowires and bend them toward the cell center (Figure 10e). To further investigate the nanowire-cell interactions, we imaged NK cells incubated on nanowires after staining their membrane with CellMask^TM^ (Figure 10f), and found that the nanowires invaginated the cell membrane, and were centripetally bent by NK cells. These findings confirmed that cells apply centripetal forces on the nanowires, and that these forces did not depend on the nanowire functionalization. We also found that the horizontal projection of the bent nanowires was about 2 µm, and used this value to calculate the magnitude of the force applied by NK cells onto a single nanowire. This force was found to be 10 picoNewton, which is the smallest force detected so far by a micro-/nano-structured surface for cell spreading.

In addition, we elucidated how the presence of nanowires, of MICA ligands, and their combination, determined the immune function of NK cells. To that end, we labelled CD107a recruited to the surface of activated cells using its fluorescent antibody (Figure 10g). We found that the percentage of CD107a-positive cells on the nanowires functionalized with MICA was substantially higher than on the control surfaces having either nanowires without MICA, or MICA without nanowires, or neither of them (Figure 10h). We also found that this difference could not stem from the available amount of MICA for cell stimulation, since we found that the surface density of MICA of flat surfaces was three times higher than that of the nanowires. Based on these results, we concluded that NK cells mechanically probe their environment, and that the high mechanical compliance of nanowires produces a physical stimulus, which, while combined with the chemical stimulus provided by MICA, triggers an enhanced immune activation of NK cells. In other words, NK cells behave as AND logic gates, whose two independent inputs—chemical and nanomechanical—determine their immune outcome, and whose logic 1 (true) output is defined as an enhanced percentage of CD107a positive cell within the overall cell population. 

It should be noted, that the nanowire morphology used in our experiments is relevant for physiological conditions. Recent reports indicated that the immune synapse is largely structured of nanosized elongated protrusions (posodomes) [98]. Also, NK cells are stimulated in vivo by interacting with dendritic cells, whose branched projections are similar in their size and shape to nanowires [99]. The role of the nanoscale topography at the interface between lymphocytes and antigen presenting cells is still unclear. Artificially created nanotopography, which mimics morphological and mechanical features of the immune synapse, enables the systematic study of the role of these features in the immune function of NK cells. Overall, the results described in this section expose what seems to be the “tip of the iceberg” of NK cell mechanosensing. Additionally, the unique nanowire-based platform that allows such a uniquely fine sensing of the mechanical activity of NK cells, can be harnessed to explore the role of nanoscale mechanical forces in the immune function of other lymphocytes such as T cells and B cells.

## 8. Future Perspective

NK cell activation is regulated by a subtle balance between activating and inhibitory signals. Understanding the molecular mechanism of this balance, and especially its spatiotemporal aspects, is an importance challenge. Overcoming this challenge can be largely aided by artificial cell environments that simultaneously control the arrangement of different receptors. However, state-of-the-art platforms used for the study of receptor clustering in NK cell, such as ligand-functionalized nanodots arrays, are designed to spatially control one receptor type. Recently, nanodots arrays functionalized with two ligands for T cell stimulation were reported [100,101], in spite of which one ligand was tethered to controllably positioned nanodots, and the second ligand was randomly distributed on the background. Nanoarrays with two or more ligands—all spatially controlled—can be implemented, through heterogeneous pattern of nanodots of different materials, which are selectively functionalized with different ligands. Realization of such nanoarrays still remains a challenge, since it requires a nanopatterning of two or more lithographic layers with sub-10 nm features and nanoscale registry. Furthermore, it requires downscaling of orthogonal functionalization approaches, which so far has been demonstrated on micron-scale patterns, to nanometric patterns. Once realized, such multifunctional nanoarrays can address basic questions regarding the spatial cross talk between interacting receptors (e.g., activating and inhibitory, or activating, and costimulatory), such as: Does their interaction and signalling integration depends on their spatial proximity? Another aspect of NK cell function, which is still largely unexplored, is their mechanical activity. State-of-the-art devices for study of cells mechanics, which have been briefly mentioned in the last section of this review, have been applied to various cells including T cells and B cells. The same experimental platforms can provide fundamental insights onto the role of mechanical forces in the function of NK cells. Finally, microfluidic devices for NK cell sorting, which are able to isolate “serial killer cells”, are essential for further studies of NK cell heterogeneity. Furthermore, isolating “serial killer cells” might be useful for an ameliorated immunotherapeutic outcome. Alternatively, these cells might be too active, and need to be discriminated to alleviate the adverse impact of immunotherapy. Currently, similar microfluidic approaches have been explored for preclinical screening of T cells in immunotherapy [102,103]. Still, these devices are used for screening, and their future therapeutic applications will likely require their combination with existing cell expansion technologies. Overall, further application of state-of the-art materials and devices for the study of NK cells, as well as the development of new materials and devices specifically tailored for NK cells, will provide important fundamental insights on their function, and allow the rational design of future immunotherapies.

## Figures and Tables

**Table 1:** Advanced materials and devices for the study and regulation of NK cells.

Type	Function	Major Contribution	General References	References on Application for NK Cells
Planar lipid bilayer	Mimic the membrane of target sell, and provide supporting 2D medium for mobile ligands	Describing receptor clustering in NK immune synapse	[25,26,27,28,29]	[30,31,32,33,34,35]
Ligand nanopatterns	Mimic the spatial distribution of discrete ligands within the membrane of target cell	Elucidating the role of antigen spatial organization in NK cell activation	[36,37,38,39,40,41,42,43]	[44,45]
Ligand Micropatterns	Mimic the clustering of ligands within the membrane of target cells	Revealing of the response of NK cells to antigen spatial segregation	[14,46,47]	[48,49,50]
Devices for physical guidance of cells	Confine or guide cells for their high-resolution microscopy	Observing the immune response of individual NK cells, and screening of NK cell sub-populations	[51,52,53,54]	[55,56,57,58,59,60,61,62,63,64,65,66,67,68,69]
Nanomaterials for the interaction with cells in 3D	Targeted delivery of controllably clustered ligands	Improving the specificity and efficacy of anti-tumor treatment	[70]	[71,72,73,74,75,76,77]
Nanodevices for the study of cell mechanical activity	Characterize forces applied by cells and its role in the cell immune function	Exploring mechanosensing in NK cells	[78,79,80,81,82,83,84,85,86,87,88,89]	[90]
